# Differential color tuning of the mesolimbic reward system

**DOI:** 10.1038/s41598-020-66574-w

**Published:** 2020-06-23

**Authors:** Kesong Hu, Eve De Rosa, Adam K. Anderson

**Affiliations:** 1 Institute of Mental Health, Nanjing Xiaozhuang University, Nanjing, China; 2 Department of Psychology, Lake Superior State University, Sault St. Marie, USA; 3 Department of Human Development, Cornell University, Ithaca, USA; 4 Human Neuroscience Institute, Cornell University, Ithaca, USA

**Keywords:** Attention, Human behaviour

## Abstract

Visual wavelengths are not only associated with the subjective experience of color but also have long been thought to regulate affect. Here we examined the attracting rewarding properties of opposite ends of the wavelength spectrum, as well as their individual variation. As reward is multifaceted, we sought convergent evidence from subjective and objective behavioral and attentional indices, as well as its neural reward system bases. On average, short (blue) relative to long (red) wavelengths were judged subjectively more pleasant and had objectively greater behavioral and attentional salience, regulating speed of simple color discriminations and perception of temporal order. Consistent with reward, these color effects were magnified following monetary reinforcement. Pronounced individual differences in color effects were related to reward but not punishment sensitivity, with blue relative to red preference associated with high relative to low reward sensitivity. An fMRI study revealed these individual differences were supported by color-dependent functional coupling between the visual cortices and mesolimbic reward circuitry. Our findings reveal the reward bases of color, demonstrating color is a potent regulator of perception, action, and neural dynamics.

## Introduction

The visual poetry of a brilliant blue sky against a smoldering yellow summer sun, vibrant red raspberries perched amongst electric new leaf green, are psychological experiences imbued with affective value. While electromagnetic waves support distinct visual experiences by the molecular mechanisms of photoreceptor transduction, what is unknown is how color has come to have broader influences beyond color perception, including modulation of behavior, perception, cognition and affect^1,2^. Indeed, color is central to marketing, brand development and design, whether to generate creative spaces in office environments or facilitate recovery in hospital settings^3^. Individuals also have unique personal connections to and preferences for color, with color thought of as a self-defining characteristic. Despite its widespread use and this subjective personal salience, the psychological mechanisms and underlying neural bases of color’s role in regulating affect and cognition remain mysterious. Color’s affective origins have variously been understood in the context of culturally determined linguistic metaphor^4^, ecological associations^5^, or biological factors^6^. While all these factors contribute to color, here we examined the differential capacity for color to recruit reward systems. Reward is multifaceted, going beyond hedonic pleasure, to include motivating behavior, regulating attentional salience, and engaging specific internal brain processes^7,8^. We propose that colors at opposing ends of the wavelength spectrum differentially engage reward, representing an underlying mechanism of general trends, and critically, contributing to individual differences in how color regulates affect behavior and attention.

Goethe long ago theorized that the human body has inherent physiological reactions to color and thus plays a central role in feelings^9^ that may arise in part from evolutionary old origins. In our original environment, the waxing and waning of light itself represented powerful selective pressures on the neural representation of color. Solar radiation contains electromagnetic wavelengths with maximum energy in the visible spectrum centered at around visible blue light and decreased toward minimal energy at long wavelength red light. Wavelength-dependent Rayleigh scatters from particulates, also changes the radiation spectrum reaching the ground, resulting in an apparent blue sky. While daylight has a prevalence of shorter blue wavelengths, this shifts toward longer red wavelengths with the darkness of night.

Wavelengths then not only influence the perception of color, but also regulate evolutionary older circadian brain functions, which regulate activity when it is safe to be active versus dormant. Our circadian systems are biologically anchored and regulated by short versus long wavelengths associated with daylight^10^. Melanopsin containing retinal ganglion cells tuned toward short wavelength blue light, directly connects to subcortical circuits^11^, regulating seasonal changes in affect^12^. The acute effect of light on melatonin suppression is strongly blue-shifted, regulating autonomic nervous system activity as well as influencing alertness, thermoregulation, and heart rate^12,13^. These wavelength-dependent changes in light across day and night then may reflect a confluence of evolutionary biological^14^ and learned ecological associations. Shorter wavelengths, e.g., blue, are also experienced as quiet and agreeable, associated with peace and tranquility, with the association with clear sky^15^. While blue may serve as a safety signal, at the opposite end of the spectrum, longer red wavelengths are thought to be stimulating and may reflect the danger of night^16^. While shorter wavelengths, e.g., blue, may elicit approach motivation, longer wavelengths, e.g., red, may be predominantly associated with avoidance. Thus, opposing ends of the visible wavelength spectrum may guide opposing affective responses, related to safety and danger. Yet, existing research reports inconsistent findings. For instance, it has been reported that red and blue were associated with happiness and sadness, respectively^17^. Even for red, some revealed that red elicited negative affect in the achievement domain^18,19^, while others showed that red yielded positive affect in the attraction domain^20^. The affective-motivational properties thus have clear context dependent exceptions.

When compared directly, red and blue have been shown to have contrasting influences on behavior and cognition. Across species, red coloration has been proposed as a sexually selected, testosterone-dependent signal of male quality and dominance^21^. Indeed, relative to those wearing blue, athletic competitors dressed in red tend to have more favorable outcomes, suggesting that red signals aggression and evokes avoidance of red in their opponents^21^ (but see^22^). Consistent with this interpretation of red as a cue for avoidance, whether with errors circled in red ink, stop signs, and other warnings, red is often associated with mistakes, alarms, danger^2^, and hostility, impairing performance in evaluative contexts^1,2^. However, while some suggested that red enhances cognitive performance as compared to blue^23,24^, others have shown the opposite^17,25^. We may expect cultural and context dependent exceptions to the role of red relative to blue, even in China, where red has highly positive cultural associations, error markings are in red, and relative to blue, red impairs student performance^26^.

An underlying relationship between color and affect may depend on some evolutionarily older cause and simultaneously be influenced in the present by culture and personal experience. Individual differences, including ethnicity, age, gender, history of depression, influence emotional responses to various colors^27,28^. Adding to this variation, color-emotional connotations are also related to lightness and chroma, and not just hue^27^. Affective-motivational properties of color thus have complex, varied bases. If there is an underlying evolutionary logic, this is also highly modifiable at the level of the individual. As such, a central aspect of our study is examining and identifying individual differences in affective responses to color, their regulation of behavior and attention, and their neural correlates. We propose that evolutionary and ecological pressures align to have differential affective and motivational tuning of the ends of the visible wavelength spectrum as well as pronounced individual differences in their expression. Individuals differ in the relative sensitivities of these largely independent neurobiological systems^29^, and thus reward or punishment. We propose that colors may come pre-prepared for associations with reward or punishment, which are in turn regulated by individual differences in sensitivity in these systems. Here we examined color regulation of affect, and its individual variation, specifically in the context of reward learning and variation in the behavioral approach system.

Reward is the attractive property of a stimulus that motivates approach behavior^30^. It evokes pleasurable feelings and provokes consummatory behaviors, which lead to behavioral reinforcement whereby behaviors become strengthened. We examined whether equiluminant colors at opposite ends of the visible spectrum are more or less readily associated with reward. We focused on more precise control of luminance, as it is a major regulator of visual salience^31^. Modelled on prior studies of color on motivation and cognition^2^, rather a systematic characterization of the effects of lightness and chroma, and hue^27^, we focused on two wavelengths at opposite ends of the visible spectrum consistently judged as red and blue. Unlike prior studies that examining what preferences people bring with them into the lab, critically we examined how color affect are altered by manipulating their association with reward, i.e., monetary reinforcement. To the degree that blue and red differ in their rewarding properties, reward association should alter red and blue’s propensity for pleasure, behavioral reinforcement, attentional salience, and differential recruitment of supporting neural reward systems^32,33^, specifically its association with a behavioral activation system (BAS)^29,34^. If color regulates reward systems, then individual differences in BAS related reward sensitivity^29^ and neural reward system tuned toward different ends of the wavelength spectrum, would in turn support how individuals differ in color salience. As such, we sought convergent evidence for the reward basis of color according to both manipulating reward, as well as, correlational trait reward sensitivity. This would provide strong new evidence of why people may both share and differ affective response to color and the widespread role of color in regulating brain systems which support affect, cognition and behavior.

## Materials and Methods

Across six Experiments, we tested whether short (blue) and long (red) wavelengths differ in connection with different facets of reward by examining (1) subjective pleasantness before (Exp 1) and following reward learning (Exp 2), (2) individual differences in reward sensitivity (Exps 2 and 4), (3) attentional incentive salience (Exps 3 and 5) and (4) individual differences in color regulation of the BAS mesolimbic reward system (Exp 6).

### Participants

Participants in six experiments were undergraduate and graduate students from Cornell University, Ithaca, NY. Sample sizes were limited at the lower end to be sufficient power based on prior studies and on the upper by availability for completing a study over a particular time period. While no strict stoppage rules were employed, data were submitted to analysis only after collection was finished for each study. All participants had self-reported normal or corrected-to-normal vision and intact color vision and were naïve to the purpose of the experiments. The Institutional Review Board (IRB) of Cornell University approved the study and all procedures and methods were conducted in accordance with its guidelines and with the Declaration of Helsinki and its latest amendments. All participants provided written informed consent before participating in the study.

### Apparatus, stimuli and procedure

Except for Experiments 1 and 2, all other experiments were conducted in a dimly lit room, and participants were tested individually. The schedule of stimulus presentation and data collection were controlled by Presentation software (Neurobehavioral Systems, Albany, CA). For experiments 3–6, each participant was given a practice run of 30 trials that were not analyzed.

The stimuli consisted of luminance matched blue, and red circles (size: 240 × 240 px^2^, 5.5 lm/ft^2^). Computer screen color was manipulated using HSL scheme: blue (160,240,120; corresponding RGB: 0, 0, 255; CIELab^35^, 29.6, 68.3, −112.0) and red (0, 240, 120; corresponding RGB: 255, 0, 0; CIELab, 54.3, 80.8, 69.9). The background was set to grey (HSL: 170, 0, 84; RGB: 84, 84, 84; CIELab, 97.6, −15.7, 93.4). Following previous work on red and blue^2,36–38^, here within a study we attempted to manipulated the hue (i.e., red vs. blue), but not chroma (saturation of color) or value (degree of darkness/lightness of the color). We have chosen to focus on selected hues, for comparison with other studies, as repeated results have failed to win complete conviction in social sciences^39,40^. An Extech Foot Candle light meter was used to measure color illuminance within the field of view. These readings do not characterize colors as printed on paper.

#### Color and affect space

In Experiment 1 (n = 20; mean age = 20, range = 18–24; 12 females), we tested affective judgments of colors on two dimensions: arousal (“not at all” to “extremely”, 1 to 7) and valence (“extremely negative” to “extremely positive”, −3 to +3). Participants viewed colors on paper on a desk and rated 3 times on these two affective dimensions. In Experiment 2 (n = 31; mean age = 20, range = 18–33, 16 females), we attempted to replicate the results of Experiment 1 and examined how variation in color preference may relate to individual differences in avoidance and approach motivational systems, using the sensitivity to punishment and sensitivity to reward questionnaire (SPSRQ)^29^. Increasing generalizability of Study 1, colors were now presented on a computer screen. Like that in Experiment 1, participants were required to rate the colors 3 times on arousal and valence.

#### Positive reinforcement

Experiment 3 (n = 19; mean age = 23, range = 19–34; 10 females) examined color interactions with reward learning, assessing which color (i.e., blue or red) was more readily behaviorally strengthened (speed and accuracy) with monetary reward. Each trial started with a fixation display for 1000 ms and was followed by a colored circle (e.g., red or blue) for 800 ms. There was a jittered 1–5 sec inter-stimulus-interval (ISI, mean: 2 sec) containing a blank screen appeared after the target display. Participants were required to indicate “red”, or “blue” with a button press. The experiment included 5 runs, and each run consisted of 36 color discrimination trials. Before the start of the experiment, participants were informed that with one of the color categories there would be an opportunity of winning monetary reward (100% contingency), while the other color was neutral (i.e., no reward). On each reward trial, participants won $0.30 if they were both accurate and fast. The reaction time (RT) threshold to determine “fast” responses was set at 650 ms. They won $0.00 during error or slow trials. On each neutral trial, participants won $0.00. Reward/neutral color pairing (red-reward vs blue-neutral; blue-reward vs red-neutral) and response mapping were counterbalanced across participants. A visual reward feedback display (duration: 1 sec) with cumulative earnings was provided at the end of each trial.

#### Attentional salience: Unrewarded colors

Experiment 4 (n = 17; mean age = 20, range = 18–24; 9 females) assessed how blue and red competed for attention, increasing salience and biasing perception of temporal order. We employed a temporal order judgement (TOJ) task with 5 × 2 × 2 design: 5 stimulus onset asynchronies (SOAs) separating first and second stimuli (8,18,38,68 and 98 ms) × 2 pairs (blue or red first) × 2 sides (first appearing stimulus was presented on the left vs. right side). There were 240 total trials, presented in two runs. In addition, we examined participants’ reward sensitivity using the SPSRQ^29^.

Each trial started with an initial fixation display for 1000 ms. Subsequently, a first color circle (either a red, or blue circle) was presented either on the left or on the right side of the fixation, and followed by a second but different color circle, which appeared on the opposite side of the first circle after a pseudorandomly determined SOA (8, 18, 38, 68, or 98 ms separating first and second stimuli). Participants were required to make a temporal order judgment, in a forced-choice manner, indicating with an appropriate button press, which was counterbalanced across participants. The response for the TOJ task was self-paced. Finally, a jittered 1–5 sec (mean: 2 sec) inter-trial interval (ITI) containing a blank screen terminated the trial.

#### Attentional salience: Rewarded colors

Employing the above task, Experiment 5 (n = 19; mean age 23, range = 19–34; 10 females) examined whether reward would modulate perceptual competition between blue and red. In this experiment, the TOJ trials were intermixed with rewarded color discrimination trials. This method is similar to that described in Experiment 3. Unlike color discrimination trials, TOJ trials were unrewarded and the responses were self-paced. The design was the same as Experiment 4: 5 (SOA separating first and second stimuli: 8,18,38,68 and 98 ms) × 2 (Pairs: blue first vs red first) × 2 (the first appearing stimulus was presented on the left vs. right side). There were 180 total TOJ trials, presented in 5 runs. Each run had 36 value reinforcement trials, together with 36 TOJ trials. Unlike color discrimination trials, feedback was not provided on TOJ performance (for similar methods, see^38,41^).

#### Functional magnetic resonance imaging: Color, affect and reward circuitry

In Experiment 6 (n = 31; mean age 20, range = 18–33; 16 females), we employed fMRI during a simple object discrimination task, with incidental processing of color. Colored circles were presented with either a rightward or leftward facing small gap. Participants were asked to discriminate the direction of the gap. Each trial started with a fixation display for 1000 ms and was followed by a colored circle (e.g., red, or blue) for 800 ms. There was a jittered 1–5 sec ISI (mean: 2sec) containing a blank screen appeared after the target display. Participants were required to indicate a “left”, or “right” by pressing a corresponding button as quickly and accurately as possible. There were 5 runs of 60 discrimination trials. Stimuli were presented on a LCD monitor form Nordic Neuro Lab at 1920 ×1080 pixels and 120 Hz refresh rate.

On each reward trial, participants either earned or saved, i.e., avoided losing, $0.30 if they were both accurate and fast. They missed the opportunity to win $0.30 (earn trial condition) or save $0.30 (save trial condition) during error or slow trials. Red and blue were mapped to potential earnings or savings of monetary reward (i.e., 100% contingency), which was counterbalanced across participants. Our analyses collapsed across the two types of reward trials. We added a yellow color (RGB, 255, 255, 0, with 5.6 lm/ft^2^) as a neutral cue, with no value reinforcement. On neutral trials, participants neither earned nor lost $0.00. Reward type x color, and response settings were counterbalanced across participants. The reaction time (RT) threshold to determine “fast” response was set to 400 ms. A visual reward feedback display (duration: 1 sec) with cumulative earnings and savings was provided at the end of the trial. We examined participants’ reward sensitivity by administering the SPSRQ following the experiment.

#### Imaging acquisition

MR data were collected using a multi-echo 3 Tesla GE Discovery MR750 (Independent receiver channel: 32). The scanning session began with a high-resolution T2-FLAIR anatomical scan (TR = 1200 ms, TE = 9.5 ms, TI = 271.2 ms, 0.3 mm isotropic voxels). Subsequently, for each functional run, BOLD EPI volumes were acquired with a TR of 2600 and TEs of 13.7, 30, 47 ms. Each volume consisted of 42 oblique slices with a thickness of 3 mm and an in-plane resolution of 3 × 3 mm (21.6 mm field of view).

### Data analysis

#### Positive reinforcement

Performance was assessed by the reaction times (RTs), and error rates. Prior to analysis, trials with reaction times of 3 standard deviations above or below the arithmetic mean were excluded for each participant. For RT analysis, the error trials were discarded.

#### Attentional salience

We probed the color effect on temporal onset judgment by analyzing participants’ hit rates between “red-blue” and “blue-red” trials (the first stimulus name appeared first physically) across SOAs. These data were then analyzed using repeated ANOVA [pair (blue-red, red-blue) x SOA (8, 18, 38, 68 and 98 ms)]. We also assessed the prior-entry effect for “Blue vs. Red”, and “Red vs. Blue”, by calculating each participant’s point of subjective simultaneity (PSS), which indicates the time interval needed by participants to perceive the two stimuli as arriving simultaneously(for method discussion, see^42^). In the present study, the Greenhouse–Geisser correction was used when the sphericity assumption was not met in ANOVAs^43^.

#### Imaging analysis

fMRI data were analyzed using tools from the AFNI software package. For the ME-ICA analysis pathway, the following preprocessing was performed on unprocessed time series data. The first 3 volumes of each functional run were discarded to account for equilibration effects. Slice time correction was applied (3dTShift). Motion correction parameters were estimated for each time point by aligning the middle TE (30 ms) images to corresponding first time point image using a rigid-body (6 parameters) alignment procedure (3dvolreg). The functional to structural co-registration parameters were estimated by registering the skull-stripped middle TE image from the first time point to the skull-stripped anatomical image using an affine (12 parameters) alignment procedure with the local Pearson correlation cost-function (3dSkullStip, 3dAllineate). Motion correction and anatomical co-registration parameters were then applied in one step (3dAllineate). A brain mask was computed from the mean image of the shortest TE (13.7 ms) time series (3dskullstrip) and applied to all images. Each image was spatially smoothed with a 5 mm FWHM Gaussian kernel within the functional mask (3dBlurInMask). As it is typical for multi-echo fMRI data, the three echoes were combined to form a single time series. These methods were described previously in^44,45^. Finally, regression analysis was performed on these data (see below). We considered three main event types in the design matrix: Blue, Red, and yellow trials. Constant, linear, and quadratic terms were included for each run separately (as covariates of no interest) to model baseline and drifts of the MR signal. To account for the signal variance related to head motion, six estimated motion parameters were included as nuisance regressors in the model (for similar methods, see^30,46^). Each run for each subject was analyzed using a fixed effect general linear model (BLOCK4 canonical hemodynamic response function).

We tested our hypotheses by interrogating activity in five color and reward-related regions of interest (ROI). As described previously^46^, At the single subject level of analysis, AFNI’s 3dROIstats function (http://afni.nimh.nih.gov/afni) was used to extract the mean activity (across voxels) associated with each contrast of interest from our priori regions of interest (ROIs). Five *a priori* brain regions were targeted that have been associated with affect and reward learning (OFC, amygdala, and ventral striatum; see^32,33^), and with color processing (V1, and V4; see^47^). OFC, V1 and V4, were created as spheres of radius 5 mm centered on peak Talairach coordinates of these regions (V1 ([0,−70,3], V4 (Left, [−27,−68,−5], Right [26,−68,−6]); OFC, (Left [−12,48,−19], right [20,45,−14]), see^32^). Left and right amygdala ROIs were defined based on anatomy using the Talairach atlas provided with the AFNI package. Additionally, Region of interest anatomical masks were created for the ventral striatum (VS) based on Nucleus Accumbens (NAcc) and extended amygdala (x: +3 to +8; y: −1 to +3, z:–1 to +6^48^). Anatomically based ROIs ensured our analyses of color and affect were unbiased. All of these ROIs were further confirmed by the meta-analysis database (http://neurosynth.org/locations). In each ROI, a representative time series was created by averaging the pre-processed time series from all the gray-matter voxels within the corresponding functionally or anatomically defined regions of interest. Finally, for each ROI, as in the regular whole-brain voxel wise analysis, deconvolution analysis was run on the representative time series data to estimate the hemodynamic response function of the main regressors.

In the present study, significance level for main statistical analyses was set at p < 0.05. Following the standard approach^46,49^, we used Pearson’s correlation as a measure of functional connectivity in the network analysis of fMRI data^50^. The 5 ×5 group level connectivity matrices were determined based on signal change (blue vs. red) from all pairs of targeted regions from all participants (for a similar approach, see^46,51)^. For all other correlation analyses in the present study, we employed iterative reweighted least squares (the robust fit function from Matlab, Mathworks, Natick, MA, USA), given that standard Pearson correlation is sensitive to even a few influential data points. As described previously^38^, significant correlations were further scrutinized via a randomization test in which the probability of each correlation was estimated non-parametrically by randomly shuffling the xy pairings (n iterations = 10,000).

## Results

### Experiment 1

**Figure 1:**
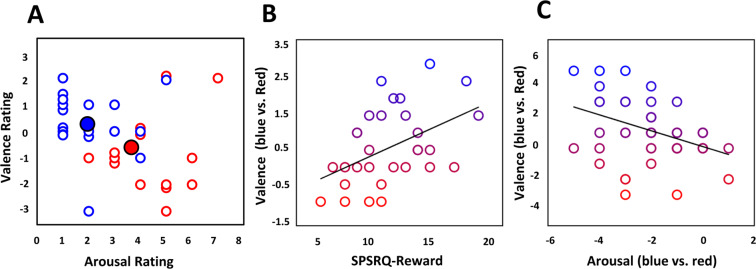
Affective representation of colors. (**A**) Two-dimensional arousal-valence ratings for red and blue. Solid symbols are means across participants; (**B**) Correlation between reward sensitivity and valence ratings; (**C**) Correlation between arousal and valence ratings.

To characterize affective responses to the ends of the visible wavelength spectrum, and their consistency across individuals, we began by simply examining where red and blue are situated in an individual’s two-dimensional affect space. Participants rated valence from −3 to +3 (extremely negative to positive) and arousal from 1 to 7 (not at all to extremely activated), to represent pleasure and activation, respectively. In valence-arousal space, individuals’ ratings of blue and red occupied were largely linearly separable in this affect space, with 87.5% correct color classification based on arousal and valence. In general, blue was more pleasant and less arousing than red (Fig. 1A). Confirming that blue has a calming and more pleasant value, arousal responses to red were significantly higher than blue, t(19) = 5.51, p < 0.001, Cohen’s d = 1.23; by contrast, blue was more pleasant than red, t(19) = 2.59, p = 0.018, Cohen’s d = 0.58. Despite large individual differences, these results are consistent with blue and red occupying distinct locations in affective space, with blue evoking, on average, more pleasure than red.

### Experiment 2

Colors have idiosyncratic influences on the individual and we found substantial individual differences in affective responses to color. If the underlying basis for these judgments is affective and related to the rewarding nature of color experience, then these individual differences may be explained, in part, by approach motivation and sensitivity toward reward^29^. We repeated the affect assessment in a separate larger set of participants (N = 31), which allowed us to assess individual differences related to two motivational systems examining approach and avoidance behavior and affect; we employed the sensitivity to punishment and sensitivity to reward questionnaire (SPSRQ)^29^. In this group, we replicated that red was significantly more arousing than blue, t(30) = 8.32, p < 0.001, Cohen’s d = 1.50, while blue was significantly more pleasant than red, t(30) = 2.55, p = 0.016, Cohen’s d = 0.60. Further, we found that reward sensitivity was related to differential valence responses between blue and red (robust fit, R^2^ = 0.147, p = 0.033). Those with the highest reward sensitivity had up to 3 times greater positive affective responses to blue over red (Fig. 1B). We confirmed the independence of reward and punishment sensitivity, robust fit, R^2^ = 0.026, p = 0.384. No relationship was found between punishment sensitivity and differential color valence (R^2^ = 0.072, p = 0.143) or red valence specifically (robust fit, R^2^ = 0.019, p = 0.457), suggesting a unique role of reward sensitivity in affective responses to color.

As blue and red occupy largely distinct portions of two-dimensional affect space, they appear to evoke qualitatively distinct forms of affective experience. Collapsing across data sets from Experiments 1 and 2, we found color dependent relationships between valence and arousal, robust fit, R^2^ = 0.168, p = 0.003 (Fig. 1C). Increasing pleasure to blue was associated with decreased arousal; conversely, increasing pleasure to red was associated with increased arousal (also see Fig. 1A). Even when blue and red similarly evoke pleasurable valence, these responses appear qualitatively distinct, representing increasing calm versus stimulation.

### Experiment 3

**Figure 2:**
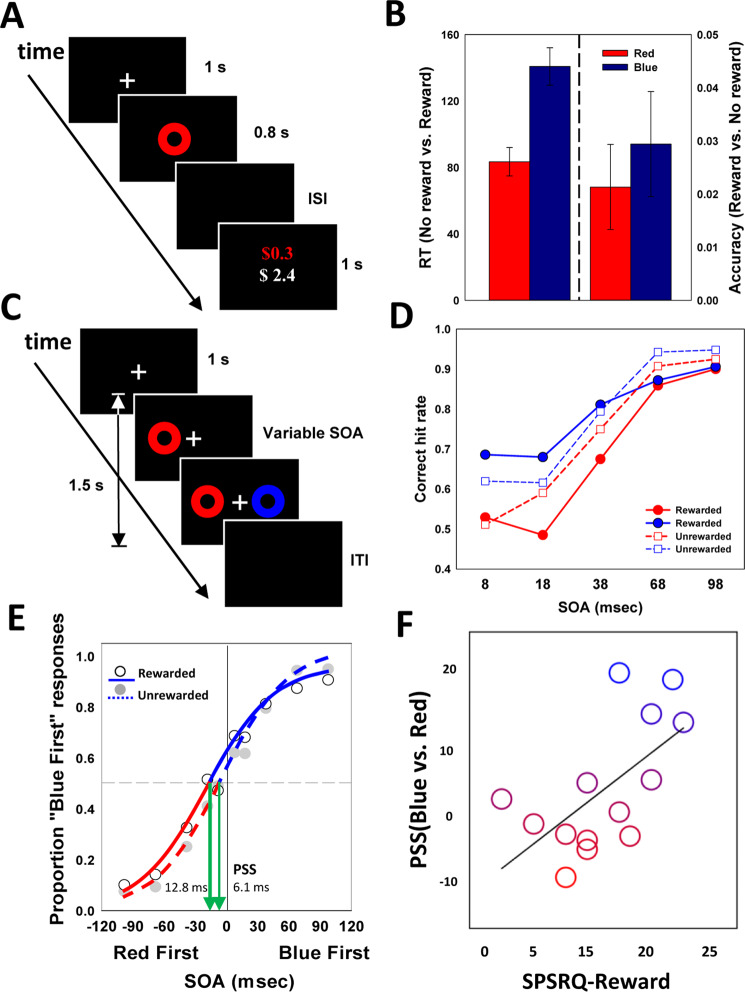
Color, reward, and attentional salience. (**A**) Reinforcement color discrimination task; (**B**) Response time and accuracy for reinforcement task (rewarded vs unrewarded). For the left panel, RT index = RT(No reward) - RT(Reward) (Left Y-axis), while for the right panel, Accuracy index = Accuracy(Reward) – Accuracy (No Reward) (Right Y-axis); (**C**) Temporal Order Judgment task; (**D**) Average hit rate proportions for each pair (blue versus red presented first, denoted by color) across SOAs; (**E**) Average proportions of “blue first” responses as a function of SOA, green arrows represent estimated point of subjective simultaneity (PSS); (**F**): Correlation between reward sensitivity and PSS.

If colors are genuinely more or less rewarding, then they should be differentially susceptible to positive reinforcement, demonstrating differential incentive salience^52^. Nineteen participants took part in an experiment where money could be earned by either red or blue circles. Participants indicated whether each circle was blue or red as quickly and accurately as possible, meeting a response time (RT) threshold of 650 ms to win $0.30 on a correct trial (Fig. 2A).

For RT analysis, error and outlier trials were excluded (<3%). First, we show that red and blue RGB values we selected reliably generated different affective responses above, were also highly reliably categorized as red and blue (>97%), even under time pressure response conditions. Across both colors, reward resulted in reinforcement of color discriminations, i.e., a facilitated behavioral response as evidenced by faster response times (RT), F(1,18) = 338.83, p < 0.001, η^2^ = 0.95, and greater accuracy, F(1,18) = 14.76, p = 0.001, η^2^ = 0.45, when colors were rewarded (Reward) compared to unrewarded (No Reward). Critically, there was also a robust interaction between reward and color for RT, F(1,17) = 342.99, p < 0.001, η^2^ = 0.95, and accuracy, F(1,17) = 15.04, p = 0.001, η^2^ = 0.47 (Fig. 2B). As shown in Fig. 2B (left panel), the RT difference (indexed by No Reward – Reward) for red was smaller than the corresponding RT difference for blue. While we did not find red or blue resulted in differential fast response times, when paired with monetary reward, blue resulted in faster performance compared to red, consistent with red and blue having differential capacity for reward. Aligning with subjective self-report, colors had objective consequences for behavior, with blue acquiring a stronger association with reward than red, enhancing speed of color discrimination.

### Experiment 4

Beyond behavioral reinforcement, the incentive salience of reward is associated with enhanced attentional salience^53^. If individuals perceive colors as differing in their rewarding properties, then colors should have an unequal priority for attention resulting in differing salience for perception. To investigate the differential attentional salience of blue and red, we examined their competition for attention, employing a temporal order judgment (TOJ) task. Red and blue circles appeared first or second across a range of stimulus onset asynchronies (SOA), ranging from 8 to 98 ms, to the left or right of fixation (Fig. 2C). Participants indicated, as accurately as possible, which side the color circle appeared first. The task had self-paced responses to emphasize perceptual salience and there was no feedback. The mean percent of correct responses were analyzed for each SOA and by which color was presented first.

Accuracy increased with increasing temporal delay between events, i.e., SOA, F(3,47) = 75.16, p < 0.0001, η^2^ = 0.83. There was a significant effect of color, F (1,16) = 4.67, p = 0.046, η^2^ = 0.23, particularly at the shortest SOA, where temporal uncertainty was greatest, t(16) = 2.95, p = 0.009, Cohen’s d = 0.72 (Fig. 2D). Compared to red, when blue was presented first it was much more likely to win the competition, consistent with a blue attentional priority. To further quantify the increased attentional salience, we estimated the point of subjective simultaneity (PSS): the temporal delay estimated between red and blue where they would be judged to be simultaneous. On average blue was perceived as arriving 6.1 ms prior to red stimuli, t(16) = 2.26, p = 0.038, Cohen’s d = 0.55 (Fig. 2E). Although this is small in magnitude of time, the effect size suggests a moderate effect size of color on attentional salience^54^. This blue-red temporal perceptual asymmetry, however, is not necessarily diagnostic of differential reward. Given that we found differences in self-reported affective responses to color were related to trait reward sensitivity, we further examined how red-blue temporal asymmetry similarly followed individual differences in reward sensitivity. Indeed, we found the magnitude of blue-shift in temporal onset increased with an individual’s reward sensitivity, robust fit, R^2^ = 0.334, p = 0.031 (Fig. 2F).

### Experiment 5

If blue and red represent differential for reward, then by manipulating reward we should be able to further increase their incentive salience and the temporal asymmetry it supports. In a separate study, we rewarded color discrimination through monetary reinforcement, whereby speeded color circle discriminations earned money ($0.30) on each trial. These monetary reinforcement trials were intermixed with TOJ trials, which were unrewarded. This source of extrinsic reward further magnified the color temporal asymmetry, with blue having an even greater accuracy advantage over red, F(2, 38) = 4.46, p = 0.001, η^2^ = 0.23, and further extended to longer SOAs between colors, SOA < 40 ms, t(18) = 2.65, p = 0.016, Cohen’s d = 0.85, (Fig. 2D). Estimating the point of subjective simultaneity (PSS), revealed that reward doubled the existing temporal priority, with blue judged on average as appearing 12.9 ms prior to red stimuli, t(18) = 2.35, p = 0.031, Cohen’s d = 0.54, (Fig. 2E), an effect size consistent with findings in how reward influences temporal judgments^55^.

### Experiment 6

If the visual experience of colors differ biologically in the capacity for reward and engagement of the behavioral activation system, then colors should be associated with altered activity within visual and reward circuits, as well as their interaction. We conducted an fMRI experiment to examine the neural bases of differential color reward as well as individual differences in their expression. We examined *a priori* (1) color sensitive visual regions and (2) affective-motivational circuitry demonstrating reward sensitivity and (3) the functional coupling between the two, in the context of reward learning. Within visual regions, we examined differential color activity in the primary visual area V1 and the higher-order cortical color region V4^47^. Within reward circuitry, we examined differential color activity in the ventral striatum (VS), amygdala, and orbitofrontal cortex (OFC). While the VS supports reward learning and its motivational states^56^, the amygdala supports value-based stimulus salience^32^, the OFC supports visual reward associations, the representation of valence and the subjective experience of pleasure^57^. Regions of interest were predefined, independently of the data under consideration, for purposes of unbiased hypothesis testing.

Color was incidental to the task: with no requirement to attend to or program behavioral responses related to color. Participants indicated whether a gap in colored circles faced left or right (i.e., a C or its reverse) as quickly and as accurately as possible, meeting a response time threshold to earn (gain) or save (avoid losing) $0.30. Gains and averted losses similarly engage the mesolimbic reward system^58^, and afforded a broader and more generalizable manipulation of reward. Both reward types yielded above 93% correct responses and thus positive outcomes (earning, 93%; saving money, 94%), consistent with a manipulation reward rather than punishment. As such, we collapsed across reward type (earn vs save) and restricted our analysis to the comparison of red and blue. Even though color was task-irrelevant, there was a trend toward faster responses to blue than red trials, t(30) = 1.91, p = 0.066, Cohen’s d = 0.34, with no significant difference in accuracy, t(30) = 0.98, p = 0.333, Cohen’s d = 0.18 or average earnings between red and blue, t(30) = 0.77, p = 0.446, Cohen’s d = 0.14.

**Figure 3:**
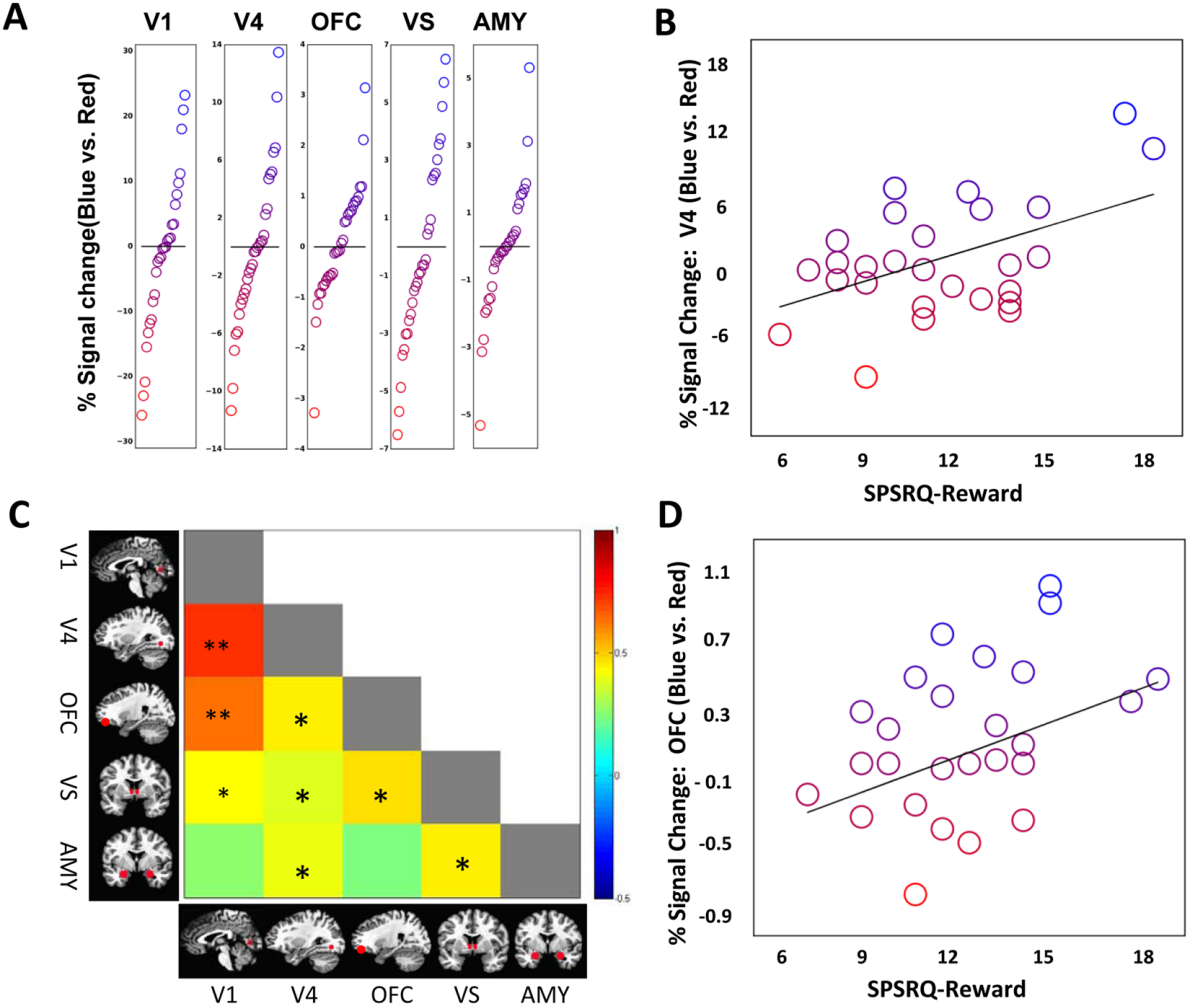
(**A**) Regional differential color responses in each participant. Note that the x-axis is rank ordered and not the same across regions, e.g., the participant with the greatest differential response to blue in V1 is not the same in the amygdala, or OFC. (**B**) Color based connectivity across brain regions of interest. Colors indicate the strength of connectivity (*p < 0.01; **p < 0.001). (**C**) Correlation between reward sensitivity and V4 differential color response. (**D**) Correlation between reward sensitivity and OFC differential color response. Primary visual cortex (V1), color cortex (V4), orbitofrontal cortex (OFC), ventral striatum (VS) and amygdala (AMY).

We next examined neural responses. There were prominent individual differences in color response to blue vs. red within each region (Fig. 3A). These variable color responses were not noise, however. When we assessed color-dependent functional connectivity between brain regions, this revealed individual differences in color tuning responses were associated with altered coupling within reward circuitry components. The VS (robust R^2^ = 0.170, p = 0.022), amygdala (robust R^2^ = 0.199, p = 0.015), and OFC (robust R^2^ = 0.255, p = 0.005), were coupled with area V4 (Fig. 3B), demonstrating reward coupling with color cortex.

While there appeared to be an equal propensity toward blue and red tuning across individuals, a major source of differential color response was individual differences in reward sensitivity. Those higher in reward sensitivity demonstrated differential blue tuning (i.e., more activated for blue than red) and those lower in reward sensitivity tended to show differential red tuning (i.e., less activated for blue than red). Within visual regions, reward sensitivity was related to color region V4, contributing to a higher blue-shift in tuning associated with higher reward sensitivity, robust R^2^ = 0.211, p = 0.016 **(**Fig. 3C**)**. Within reward regions, greater reward sensitivity was associated with greater blue-shift in each the OFC (robust R^2^ = 0.178, p = 0.027), amygdala (robust R^2^ = 0.206, p = 0.010), and at trend level, in the VS (R^2^ = 0.133, p = 0.062) (Fig. 3B,D). There was no evidence of a relationship between color tuning and punishment sensitivity, all R^2^ < 003, smallest p = 0.770).

**Figure 4:**
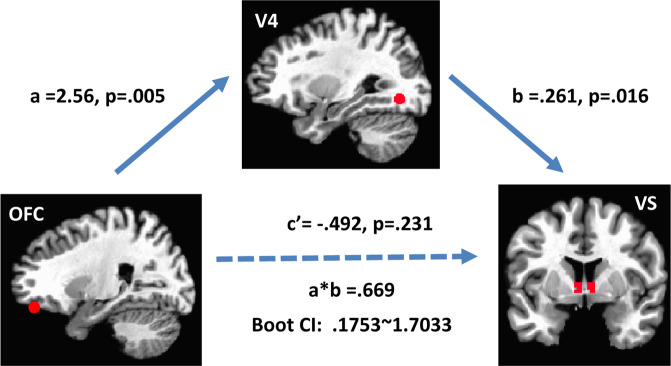
Mediating role of color in reward circuit interactions. Mediation analysis examined role of color response in color cortex in reward system interactions. The dotted line indicates the adjusted path coefficient c’ between OFC (IV) and VS (DV) was reduced to nonsignificance after V4 mediator was taken into account. The letters a, b, a*b and c’ refer to estimated path coefficients. Color cortex (V4), orbitofrontal cortex (OFC), ventral striatum (VS). Boot CI is the bootstrapped confidence interval.

To examine the central role of V4 cortex in color reward, we conducted two mediation analyses (for a similar approach, see^46,59–61^). We chose the VS as the dependent variable, given its role in reward learning and motivation^33^. We found that V4 mediated color-specific coupling between the OFC and the VS (Fig. 4). A bias-corrected 95% bootstrap confidence interval (CI) for the indirect effect (a_1_*b_1_ = 0.669) based on 10,000 samples was entirely above zero (0.1753~1.7033), such that the total color effect between OFC and the VS was dependent on V4. To address the psychological and motivational dimension of color based affect, we tested an additional mediation model with reward sensitivity. We found V4 mediated the association between VS response and reward sensitivity. A bias-corrected 95% bootstrap CI for the indirect effect (a_2_*b_2_ = 0.209) was entirely above zero (0.0124 ~ 0.5037). These results suggest that color-based affect is related to the neural bases of color perception. In particular, V4 is central to the expression of color based reward, both in terms of orchestrating interactions within reward circuits and individual differences in reward experience. In sum, these findings support that affective, motivational and perceptual representations underlie individual differences in the experience of color affect.

## Discussion

Color preference is often an identity defining feature, where individuals often describe feeling connected to some colors, but not others. The present studies examined the role of color in both neutral and reward reinforcement contexts to address the inherent and context-dependent affective properties of opposing ends of the visible wavelength spectrum. We found large individual differences in color tuning, at the level of affect, behavior, and reward circuitry. The brain and behavior effects of color were further associated with reward sensitivity. These findings suggest that colors have unique effects on an individual’s brain dynamics and performance that go well beyond the perception of color. In addition to these individual differences, there was a general blue-shift in the reward properties of color, which was strengthened by intrinsic reward sensitivity and extrinsic reward reinforcement. Contrary to the adage of seeing through rose-tinted glasses, here we show blue is more readily associated with a rewarding perceptual experience than red.

According to color-in-context theory^14^, color effects are rooted in the repeated pairing of color and particular concepts, messages, and experiences, and in this way, the mere perception of the color may evoke meaning-consistent affect, cognition and behavior. Blue is often metaphorically associated with calmness, comfort, and peace, and is often used in healthcare settings, like wall color in hospitals^3^. However, in other contexts blue may signal negative emotions, like sadness and disgust^17^. Red symbolizes fire, danger and mistakes, a metaphor for war, rage, and anger, evoking hostility; however, in other contexts red is associated with love and attraction^1,9^. While blue increases pleasant value judgments, which may lead to approach motivation, red has a larger influence on arousal, which is relevant to both avoidance and approach motivation, depending on context. Here we examined colors in the context of reward, where in the great majority of trials, participants performed correctly and received monetary reinforcement. In this context, red should have been associated with good luck and fortune, enhancing its positive value. Despite this context, on average, red acquired less incentive salience than blue and was less associated with individual differences in reward sensitivity and recruitment of reward circuity. The manipulation of reward, may have also contributed to our finding of a relationship with color and reward and not punishment sensitivity. It is possible, as we found here, that there is a unique relationship between the behavioral activation system, reward sensitivity, and color. However, without manipulating punishment, we can assert that punishment sensitivity, and the associated behavioral inhibition system, does not play a role in individual differences in color.

Aligning with a physical dimension of light, pleasantness largely decreases with increasing wavelength, with blue as one of the most pleasantly experienced colors^62^. There are many ecological associations that may account for these preferences^5^ that align with other likely natural selective biological factors. Blue sky and water indeed represent ecological positive associations, yet these also rest on the physics of light and biological adaptations to it. For instance, Melanopsin-based receptors are tuned to blue wavelengths and have strong physiological effects, including hypothalamic circadian and affective regulation^13^, suppressing endogenous melatonin and enhancing cognitive performance, affording a potential direct biological influence of short wavelengths, and blue color, related to daylight. As such, looking at the world through colored lenses has more than metaphorical associations. It may directly regulate brain dynamics^10,11^, mental states and cognitive performance^2^.

Our results should not be taken to imply that blue is a general enhancer of cognitive function. Red has been shown to enhance certain types of problem solving^2^ and is associated with enhanced sports performance and the chance of winning^21^. While it is not clear how prior findings on the performance enhancing properties of red reflect altered attention, color may have distinct effects on selective attention. Consistent with the effects of low arousal positive affect on reducing selective attention and enhancing creative thinking^63^, blue may hurt focus, while red may enhance focus and executing of well-practiced strategies. Just like emotions and their regulation of perception and cognition^63,64^, colors may help or hurt depending on the context and the specific task at hand. Color effects are context specific and the “context” should be considered not only in terms of domain, but also in terms of culture^25,65^. Together, there is no one optimal emotion or color.

Participants’ reward sensitivity predicted differential color responses in V4, suggesting color reward effects are related to the experience of color. Reward circuitry, including the ventral striatum, medial OFC and amygdala^32,56,57^ not only demonstrated functionally coupled color tuned responses, but these responses also correlated with differential color responses in V4. Mediation analyses demonstrated that differential color activity in V4 was central to orchestrating interactions between reward circuit components. Together, these findings suggest color processing in V4 is differentially associated with reward sensitivity and reward circuitry, providing an account of how color is imbued with affective properties.

Our previous findings suggest that affect may function as an abstract feature across the senses in the OFC^57^ as well as sensory specific dimensions, whether in vision or taste^57,66^. As proposed by Wundt^67^, this renders affect a core perceptual attribute of all sensory experience^68^. In this way, affect is bound to specific sensory events, enhancing their impact and vividness^69^. That we find evidence of the rewarding properties of color in both the OFC and visual cortical area V4 may indicate the existence of abstract and sensory specific components of color affect^66^. While abstract representations of affect may mediate connections between sensory systems, as found between color and music^70^, the involvement of area V4 may support a more direct experience of color as rewarding.

There are several limitations to consider in the present results. Color perception is highly dependent on lighting and other conditions. As were primarily concerned with matching luminance to match perceptual salience, we used a Foot Candle light meter to measure color illuminance within the field of view, but not a colorimeter. Lighting was not precisely controlled and we were not able to conduct colorimetric analyses across our different methods of presenting color stimuli. For instance, we attempted to selectively manipulate the hue, but not chroma or value, yet they still varied unintentionally in presentation^35^, and across studies. This is particularly the case when we moved from behavioral testing to the projector system used for fMRI. Despite our inability to precisely characterize hue and its perception, our data provided evidence that the color patches were judged accurately as red and blue. We also selected only two colors, at opposing ends of the visible spectrum, contrasting blue and red following a prior approach that explored the effect of color on cognitive task performance^2^. We note that the temporal order judgment task itself has this unique limitation^54^ in that only pairwise comparisons are possible^71^. As such, our findings may reflect responses to blue or red, or both colors. Indeed, the limiting of comparisons to blue and red is itself a context in which the colors are interpreted, which may limit the generalizability of the results to other color contexts. Finally, given that culture plays a role in determining the kind of situations in which mixed color-emotions are expected, future study needs to determine if our findings are generalized across eastern and western worlds.

Unlike sweet and bitter taste or caressing touch and pain, which have specialized receptors and also serve as primary reinforcers and punishers, visual stimuli are thought to require ecological associations and developmental experience to acquire affective properties^66^. Nevertheless, it is tantalizing to think that specific photoreceptor wavelengths may have been naturally selected based on navigating pleasure and pain. Such biases in color tuning may be readily overtaken by ecological factors and social learning. Indeed, despite a blue-shift in reward processing, we find large individual differences in neural color tuning and show with reinforcement learning, color incentive salience is malleable, influenced by even recent exposure and association with reward. Through biology and experience, each individual’s brain may be uniquely tuned to different wavelengths. While specific regions of the visible spectrum may have a tendency toward reward, in each individual this is expressed differently, embodied in reward circuits interactions with the visual representation of color. Although the precise wavelengths may differ in each individual, our results suggest visible wavelengths go beyond the experience of color, regulating how the brain supports attention, action and reward.

## Data Availability

The data that support the findings of this study are available from the corresponding authors upon request.
